# Using Theory of Planned Behaviour to Assess the Determinants of Uptake of Modern Family Planning among Women of Reproductive Age in Rural Settings of Morogoro, Tanzania; A Cross-Sectional Study

**DOI:** 10.24248/eahrj.v8i3.805

**Published:** 2025-01-30

**Authors:** Dionisia Danda, Erick Donard Oguma, Fabiola Vincent Moshi

**Affiliations:** a Department of Clinical Nursing, School of Nursing and Public Health, University of Dodoma, Dodoma Tanzania

## Abstract

**Background:**

Modern family planning is considered one of the most cost-effective health intervention to improve the well-being of women and newborns. Little is known about the influence of the theory of planned behaviour on the uptake of modern family planning. Therefore, the study aims to address the existing gap in using the theory of planned behaviour to explain the uptake of modern family planning in rural settings of Morogoro, Tanzania.

**Methods:**

The community-based analytical cross-sectional study was conducted at Malinyi District in rural Morogoro, in June to July 2022. A multistage sampling technique was used to select 421 women of reproductive age. A structured questionnaire adapted from previous studies was used as the data collection tool. The determinants for the uptake of modern family planning were analyzed using bivariable and multivariable binary logistic regression model. The final results, which were statistically significant from the regression analysis model were presented using Adjusted Odd’s Ratio (AOR), Confidence Interval (95% CI), and *p*-value <.05.

**Results:**

The majority of women of reproductive age, 328 (78%), were using modern family planning methods, with more than half of respondents (54.1%) using implants. Depo Provera (29.1%) and the pill (14.4%) were the second and third most frequently used methods. Only 4.9% reported IUCD and 2.8% condom use. The majority of the women, 342 (81%), had a positive attitude, positive subjective norms 289 (68.6%), positive perceived behaviour control 388 (92%), and high intention on uptake of modern family planning 61 (85.7%). The women with a positive attitude on modern family planning uptake (AOR 2.307: 95% CI, 1.243 to 4.281) and positive perceived Behavioural control (AOR 6.015: 95% CI; 0.017 to 2.569) were more likely to be significantly associated with increased uptake of modern family planning. Those with high intention on uptake of modern family planning (AOR 0.038; 95% CI; 0.018 to 0.080) were less likely to be significantly associated with increased uptake of modern family planning.

**Conclusion:**

Positive attitude and high perceived behavioural control have a direct positive effect on the uptake of modern family planning. The family planning education-based programs could be offered to the community, particularly in rural areas, to maximise the community awareness and uptake of modern family planning. The theory of planned behaviour could predict intention to use but not the actual utilisation of modern family planning. A combination of theoretical models may be required to understand additional external elements that may influence the utilisation of modern family planning.

## BACKGROUND

Globally, an estimated 1.9 billion women are thought to be in the world’s reproductive age group between 15–49 years. About 1.1 billion women of reproductive age (15–49 years) have a need for family planning. ^1^ Among 1.1 billion, an estimated 874 million using modern contraceptive methods, 92 million use traditional methods, and 164 million have an unmet need for family planning.^1^ The prevalence of uptake of modern family planning among women of reproductive age is accounted for by more than 80% in Australia and New Zealand, Eastern and South-Eastern Asia, Europe and Northern America, and Latin America and the Caribbean. The sub-Saharan Africa and Oceania regions, excluding Australia and New Zealand, were regions with low prevalence of modern contraceptive use and accounted for 56% and 52%, respectively.^1^ It seems to be a remarkable difference in the strategies employed to sensitise and promote the uptake of modern family planning among women of reproductive age in developed and developing countries.

The use of modern family planning methods among women of reproductive age in Tanzania is expected to reach 60% by 2025. However, the current trend of using modern family planning methods is at 31%, which is below the expected threshold.^2^ In Morogoro region, the prevalence of modern family planning uptake is 38.9% greater than the national prevalence.^2^ In the East African Community, the contraceptive prevalence of any method is higher in Kenya as compared to Tanzania (38%), Uganda (41.8%), Rwanda (53.2%), Burundi (28.5%), and South Sudan (4%) (World Bank, 2021). Fear of side effects, misconceptions, fertility desire, gender preferences of children, ethnicity, religion, lack of information, unavailability of the preferred method, lack of trained personnel, and discouragement from a partner were the major barriers to modern family planning uptake.^3,4,5,6^

Modern family planning services are widespread and considered one of the most cost-effective health interventions to improve the well-being of women and children.^7^ Effective use of family planning will help to decrease maternal morbidity and mortality, neonatal death, and facilitate economic growth and reduce overpopulation.^8^ Family planning was believed to have lowered maternal death by 6 to 60% in various nations, newborn mortality, and abortion rates, particularly unsafe abortions.^9^ The effectiveness of family planning to prevent maternal fatalities is considered 43.3%.^10^ The use of modern contraceptives in 2017, prevent an estimated 308 million unintended pregnancies. Addressing all women’s need for family planning would help to prevent an additional 67 million unintended pregnancies annually. ^11^

The lack of financial resources, access to information, and access to healthcare facilities are the major barriers to the uptake of modern family planning reported in previous studies.^12^ The literature indicates that, knowledge and attitude toward modern contraceptive methods and institutional factors such as availability of supplies, long waits or stays for care, and insufficient medical staff and qualifications affect the uptake of modern family planning.^3^ It is also noted that knowledge and attitude toward uptake of modern family planning services are affected by uneven reproductive preferences, inadequate education, and false impressions about the side effects of contraceptives.^13^

The theory of planned Behaviour it has been playing the crucial role in predicting the Behavioural intention. According to this theory, the behavioural intention, or the likelihood of behaviour, is influenced by three domains, which are attitude, perceived subjective norm, and perceived behavioural control. The culmination of these three constructs leads to intentional and behavioural changes. ^14^ Little is known about the influence of domains of the theory of planned behaviour on the uptake of modern family planning services by women of reproductive age in the context of Tanzania, specifically in rural Morogoro. Therefore, this study intended to use the theory of planned behaviour to assess the determinants of the uptake of modern family planning among women of reproductive age in the Morogoro region.

## METHODS

### Study Setting and Study Design

A community-based cross-sectional analytical study was conducted from June to July, 2022 in Malinyi district, Morogoro region, which is located in the eastern part of mainland Tanzania. According to the 2022 census, the region had a population of 3,197,104, and Malinyi district had a population of 225,126. Morogoro region has a total of seven districts, namely Gairo, Mvomero, Kilosa, Morogoro rural, Kilombero, Malinyi, and Ulanga. ^15^

Geographically, the region is bordered by the Tanga region to the north, the Pwani region to the east, the Ruvuma region to the south, and the Iringa and Dodoma regions to the west ^16^. There are 463 medical facilities in the region, including 388 dispensaries, 57 health centres, and 17 hospitals. Every healthcare facility is qualified to offer family planning services. The region’s fertility rate is 3.9, the average number of children per woman is 5.2, and maternal mortality is 415/100,000 live births.^16^

### Inclusion and Exclusion Criteria

The study included all women of reproductive age, 15 to 49 years of age, living in the Malinyi Morogoro region and who voluntarily agree to participate in the study were included. However, women of reproductive age who were reported to be mentally ill, seriously sick and those who refused to participate were excluded.

### Sample Size

The minimum sample size was computed using the Cochran’s formula. The reasoning for choosing this formula, because the Cochran’s formula determines the sample size for an infinite population.


$$\text{N}=\frac{{\text{Z}}^{\text{2}}\times \text{P}\times \text{q}}{{\text{E}}^{2}}$$

Where; N= Minimal sample size.

Z=Standard normal deviation set at 1.96 (corresponding to confidence level of 95%)

E = Marginal error is 5%

q = (1-P)

P= Proportion of the estimated women of reproductive age in Morogoro who are using modern family planning (47% per TDHS 2016) ^16^

Therefore, Minimal sample size = 421

The minimum sample size in this study was 421 women of reproductive age (15–49 years).

### Sampling Procedure

A multistage sampling method was used to select the study participants in this study; a simple random sampling method was used to select 2 districts out of 7 by the lottery replacement method. On average, each district had 4 divisions. Therefore, 1 division in each district was randomly selected, making a total of 2 divisions. Two wards out of 5 were randomly selected, making a total of 4 wards. Two villages were randomly selected from each of the four wards, making a total of 8 villages. Households with participants meeting the study criteria were identified, listed, and approached with the help of community leaders. All the participants meeting the selection criteria were interviewed.

### Data Collection Methods and Tools

#### Data Collection Methods

The principal investigator and four trained research assistants conducted data collection within the selected wards and research assistants were community health works. Data collection procedures were conducted in an unoccupied separate place to ensure the privacy and comfortability of the study respondents who was seated on separate chairs to prevent sharing of responses. All participant’s information was obtained through interviewer administered questionnaire. The rapport was first built by the interviewer before any data was collected. The participants were requested to participate after an introduction explaining the study’s goal. Participants’ privacy and freedom to participate in the study were guaranteed by the researcher. Before collecting any data, we obtained the participant’s verbal and written consent.

#### Data Collection Tools

A structured questionnaire adapted from previous study was used to collect data, and the questions were modified to fit the study. ^17^ Tool was prepared in English language, then it is translated into Swahili language to make it user-friendly to the participants. The back translation approach was used to ensure the accuracy of translated questionnaire. The final version of the questionnaires was organized into of three parts with a total of 38 items. To ensure the content validity, a questionnaire was reviewed by a panel of two experts, to ensure it fully represents the construct. Then, a pilot study was conducted to pre-test the questionnaire using 10% of sample size, leading to minor modifications based on feedback to improve clarity and cultural relevance. The reliability test was conducted and the Cronbach’s alpha was above 0.7 which is acceptable.

### Research Variables

#### Independent Variable

The independent variables in this study were the domains of theory of planned behaviour, these were;

##### Attitude Towards Modern Family Planning

The attitudes towards modern family planning were measured in 12 items measured in a 5-point Likert scale. The total score was 60 points, whereby the minimum score was 33, the maximum score was 60, and the mean score was 44 (± 4.170 SD). Since the data were normally distributed, later, the mean score was used as a cut-off point to categorise the attitude on modern family planning uptake among women of reproductive age.

##### Perceived subjective norms toward modern family planning uptake

The perceived subjective norms are measured with 6 items (partner support, pressure from friends, pressure from healthcare providers, community leaders, and parents). Six statements were used to measure perceived subjective norms. This independent variable was assessed by using a 5-point Likert scale ranging from strongly disagree 1, disagree 2, neutral 3, agree 4, and strongly agree 5.^18,19^ All these variables were measured by the ordinal scale and analysed descriptively as frequency and proportion. The total score was 30 points, whereby the minimum score was 13, the maximum score was 29, and the mean score was 21 (± 2.217 SD). Since the data were normally distributed, later, the mean score was used as a cut-off point to categorise the perceived subjective norms on modern family planning uptake among women of reproductive age. The respondents who scored greater than or equal to the mean value were categorised as having positive subjective norms on modern family planning uptake, and those who scored less than the mean value were categorised as having negative subjective norms on modern family planning uptake.

##### Perceived behaviour control

The perceived behaviour control was measured by 10 items. As an independent variable, perceived behaviour control was assessed by using a 5-point Likert scale ranging from strongly disagree (1), disagree (2), neutral (3), agree (4), and strongly agree (5). ^18,19^ All these variables were measured by the ordinal scale and analysed using descriptive statistics and reported as frequency and proportion. The total score was 50 points, whereby the minimum score was 10, the maximum score was 50, and the mean score was 41 (± 4.733 SD). Since the data were normally distributed, later, the mean score was used as a cut-off point to categorise the perceived behaviour control on modern family planning uptake among women of reproductive age. The respondents who scored greater than or equal to the mean value were categorised as having positive perceived behaviour control on modern family planning uptake, and those who scored less than the mean value was categorised as having negative perceived behaviour control on modern family planning uptake.

##### Intention behaviour

The intention behaviour towards modern family planning uptake among women of reproductive age was evaluated by requesting whether the coming twelve-month intent to use modern family planning. Each item asked was dichotomised into (1) Yes and (0) No. The Yes responses were counted as modern family planning use intent, and the No, indicated no intent.

**Socio-demographic characteristics** were evaluated by observing the characteristics of the study population, i.e., age, education level, occupation, marital status, marriage duration, number of parity (children in the family), and average monthly income. Ten multiple-choice questions were used to assess this section.

#### Dependent Variable

The uptake of modern family planning was the dependent variable. Women of reproductive age were asked whether they had used modern family planning for the past twelve months. The response was dichotomised into (1) Yes and (0) No.

### Data Analysis

Data was analysed using SPSS version 26. Before the analysis process, data was cleaned, measured for normality, organised, and converted into an analysable format. Descriptive analysis was done to establish frequencies and percentages. Bivariable and multivariable analyses were done using binary logistic regression analysis as an inferential analysis model to assess the association of independent variables (socio-demographic characteristics and TPB domains on modern family planning uptake). The final results, which were statistically significant from the regression analysis model, were presented using Adjusted Odd’s Ratio (AOR), Confidence Interval (95% CI), and *p*-value < 0.05.

### Ethical Considerations

The ethical approval was obtained from the University of Dodoma, Institutional Research Review Committee on 13 th June, 2022 with (Ref. No. MA.84/261/02/). Permission for data collection was sought from the Regional Administrative Secretary (RAS) of Morogoro region on 10 th June, 2022 with (Ref. No. AB.175/245/01|43). Each participant was asked to sign informed consent form before participation, and were free to withdraw from participation at any time. Participants’ values, integrity, and dignity were safeguarded throughout the study period. The anonymization and de-identification were used to ensure participants’ confidentiality and personal privacy. Additionally, the participants’ data were secured and protected against the access from unauthorized person.

## RESULTS

### Sociodemographic Characteristics of Study Respondents

A total of 421 women of reproductive age were enrolled in this study. The respondents’ age ranged from 18 to 49 years, with the mean age of 31.22 ± 8.549. The majority, 311 (73.9%), were married, and about 158 (37.5%) of respondents were aged between 35 and 49 years. Approximately 269 (64%) women had between one and three children, while 91 (21.6%) had four to five children, and only 61 (14.5%) had more than five children. Likewise, the study involved women with varying education statuses, among whom 11.6% did not attend school, and 66.5% and 21.4% reached primary and secondary education levels and above, respectively.

The study involved women with varying occupations, among whom 35 (8.3%) were housewives, 280 (86.2%) were farmers, 15 (3.6%) were employees, and 8 (1.9%) combined formal employment and business. More than 80% (339) of participants had a monthly income of less than 100,000 Tanzania shillings. (Table 1).

**Table 1:** Socio-Demographic Information of Women of Reproductive Age (N=421)

Variables	Frequency (n)	Percent (%)
Age category (Years)		
18-24	111	26.4
25-34	152	36.1
35-49	158	37.5
Number of Parity		
≤ 3	269	63.9
4-5	91	21.6
> 5	61	14.5
Marital status		
Single	73	17.3
Married	311	73.9
Divorced	26	6.2
Widowed	11	2.6
Religion		
Muslim	126	29.9
Christians	295	70.1
Maternal education status		
Informal	49	11.6
Primary	280	66.5
Secondary	92	21.9
Maternal Occupation		
Housewife	35	8.3
Farmer	363	86.2
Employed	15	3.6
Business	8	1.9
Partner occupation		
Employed	21	5.0
Farmer	286	67.9
Business	7	1.7
Not applicable	107	25.4
Years in marriage		
1-5	108	25.7
6-10	111	26.4
11+	95	22.6
Not applicable	107	25.4
Average monthly income in TZS		
< 100,000	339	80.5
100,000-500,000	57	13.5
> 500,000	25	5.9

### The Attitude towards MFP among Women of Reproductive Age

The study findings revealed that 81.2% of respondents had positive attitudes and 19.8% had negative attitudes among women of reproductive age on modern family planning uptake methods. Findings indicated that modern family planning was primarily used for child spacing 331 (78.6%) and managing their own health 334 (79.3%). However, the majority, 198 (47.0%), of participants indicated that they did not use condoms as a modern family planning method, despite the additional advantage offered by condoms in the prevention of sexually transmitted diseases. The overall findings in Table 2 indicate that the majority of women had positive attitudes towards modern family planning. (Table 2).

**Table 2:** The Attitude Towards Modern Family Planning Uptake Among Women of Reproductive Age (N=421)

Items	S/Disagree n (%)	Disagree n (%)	Neutral n (%)	Agree n (%)	S/Agree n (%)
If I use modem family planning, I am doing something good	0 (0.0)	4 (1.0)	12 (2.9)	221 (52.5)	184 (43.7)
If I adhere to the modem family planning method, I am doing something good	1 (0.2)	5 (1.2)	9 (2.1)	288 (68.0)	118 (28.0)
Modem family planning method has minor side effects	12 (2.9)	61 (14.5)	27 (6.4)	273 (64.8)	48 (11.4)
If I use modem family planning, I am not against my culture	0 (0.0)	3 (0.7)	6 (1.4)	249 (59.1)	163 (38.7)
If I use modem family planning, I am not against my religious	0 (0.0)	3 (0.7)	13 (3.1)	324 (77.0)	81 (19.2)
If I use modem family planning, I ensure my own health	0 (0.0)	9 (2.1)	6 (1.4)	334 (79.3)	72 (17.1)
The use of modern family planning method ensures good economic status	7 (1.7)	98 (23.3)	25 (5.9)	247 (58.7)	44 (10.5)
If I use modem family planning method, I can space my children and-hence I will have health children	1 (0.2)	3 (0.7)	5 (1.2)	331 (78.6)	81 (19.2)
If I use modem family planning, I strengthen my relationship with-my husband	8 (1∙9)	23 (5.5)	46 (10.9)	306 (72.7)	38 (9.0)
If I use modem family planning method such as condom protect-sexually transmitted diseases	43 (10.2)	198 (47.0)	98 (23.3)	70 (16.6)	12 (2.9)
Use of modern family planning decrease poor birth outcome	0 (0.0)	14 (3.3)	22 (5.2)	361 (85.7)	24 (5.7)
Waiting for modern family planning service care in the health-facilities is not long	6 (1.4)	12 (2.9)	18 (4.3)	353 (83.8)	32 (7.6)

### The Perceived Subjective Norms towards Modern Family Planning among Women of Reproductive Age

The results showed that approximately 68.6% (n=289) of respondents had high perceived subjective norms and 31.4% (n=132) low perceived subjective norms about adopting modern family planning in women of childbearing age. Data analysis also shows that pressure exerted by healthcare providers had a major impact on MFP uptake among women of reproductive age, 97.3% (n=410). However, community leaders 39.2% (n=165) did not support the use of women, as the majority of them opposed the use of MFP 60.8% (n=256). Cultural leaders 16.8% (n=71) emerged as a factor not supporting the inclusion of MFP as part of the subjective norm construct. (Table 3).

**Table 3:** The Perceived Subjective Norms on Modern Family Planning Uptake Among Women of Reproductive Age (N=421).

Items	S/Disagree n (%)	Disagree n (%)	Neutral n (%)	Agree n (%)	S/Agree n (%)
My community leaders think I should use modern family planning.	43 (10.2)	192 (45.6)	21 (5.0)	137 (32.5)	28 (6.7)
My cultural leaders think I should use modern family planning	3 (0.7)	337 (80.0)	10 (2.4)	54 (12.8)	17 (4.0)
My husband thinks I should use modern family planning	42 (10.0)	2 (0.5)	26 (6.2)	19 (4.5)	332 (78.9)
My peers think I use modern family planning	7 (1.7)	0 (0.0)	7 (1.7)	133 (31.8)	273 (64.8)
My health care provider thinks I should use modem family planning	1 (0.2)	2 (0.5)	8 (1∙9)	148 (14.2)	262 (62.2)
My religious leader thinks I should use modem family planning	47 (11.2)	366 (86.9)	5 (1.2)	2 (0.5)	1 (0.2)

### The Perceived Behavioural Control on Modern Family Planning Among Women of Reproductive Age

Study results showed that 92% (n=388) of women of reproductive age had positively perceived behavioural control in modern family planning, and 8% (n=33) had negatively perceived behavioural control. Most women of reproductive age, 83.4% (n=351), are confident to consult modern family planning methods in healthcare facilities after being able to stop taking or using MFP methods whenever they want, 81.5% (n=343). MFP use is primarily to prevent unwanted pregnancies: 59.9% (n=252), facilitated by the associated low cost: 69.6% (n=293) of sourcing needed items. Despite the consensus of the majority about the willingness to talk to their loved ones about MFP 79.3% (n=334), some of them are reluctant to talk to their loved ones about MFP 4.3% (n=18), which is the highest percentage in the category “Disagree.” (Table 4).

**Table 4:** Perceived Behavioural Control on the Modern Family Planning Uptake Among Women Of Reproductive Age (N=421)

Items	S/Disagree n (%)	Disagree n (%)	Neutral n (%)	Agree n (%)	S/Agree n (%)
For me to use available modern family planning methods is-Simple and I can do so.	10 (2.4)	11 (2.6)	0 (0.0)	306 (72.7)	94 (22.3)
I feel confident for me to be counseled on the uptake of-modern family planning	5 (1.2)	6 (1.4)	2 (0.5)	300 (71.3)	108 (25.7)
I feel confident that modern family planning will prevent-unwanted pregnancy	4 (1.0)	2 (0.5)	3 (0.7)	252 (59.9)	160 (38.0)
I have the confidence to suggest to my friends to use modernfamily planning for child spacing	3 (0.7)	10 (2.4)	4 (1.0)	327 (77.7)	77 (18.3)
I am confident to ask about modern family planning methods-in a health institution.	4 (1.0)	9 (2.1)	4 (1.0)	351 (83.4)	53 (12.6)
I feel the cost of modem family planning methods is easy to overcome.	3 (0.7)	2 (0.5)	6 (1.4)	293 (69.6)	116 (27.6)
I am confident to talk about family planning methods with my friends	4 (1.0)	15 (3.6)	2 (0.5)	338 (80.3)	62 (14.7)
I can talk about family planning methods with relatives	4 (1.0)	18 (4.3)	2 (0.5)	334 (79.3)	63 (15.0)
I can stop taking/using FP methods whenever I want to.	3 (0.7)	7 (1.7)	6 (1.4)	343 (81.5)	62 (14.7)
I can stop taking/using FP methods at my own convenience	3 (0.7)	7 (1.7)	6 (1.4)	331 (78.6)	73 (17.3)

### Intention to Use Modern Family Planning Methods among Women of Reproductive Age

The study results showed that most of the 361 respondents (85.7%) had a high intention to use various types of modern family planning methods. And about 14.3% (n=60) of women of reproductive age had a low intention to use modern family planning in women of reproductive age.

### Associations between Socio-Demographic Characteristics, TPB Domains, and Modern Family Planning Uptake.

A binary logistic regression analysis was calculated for variables such as marital status, marriage length, occupation, attitude, behaviour control, subjective norm, and intention. The results show that for marital status, the category of married (OR 0.366; *P* < .001) and intent (OR 0.041; *P* < .001) were significantly associated with the inclusion of MFP before adjustment for confounders. Attitude, subjective norms, and behavioural control were significantly associated with MFP uptake with (OR 4.212; *P* < .001), (OR 1.967; *P* < .043), and (OR 9.306; *P* < .001), respectively. After adjusting for confounders, attitude (AOR 2.307; 95% CI; 1.243 to 4.281), intention (AOR 0.038; 95% CI; 0.018 to 0.080), and perceived behavioural control (AOR 6.015; 95% CI; 0.017 to 2.569) were significantly associated with MFP uptake. (Table 5)

**Table 5:** Association between Socio-Demographic Characteristics, TPB Domains and Modern Family Planning Uptake

Variable	95% CI				95% CI			
	OR	Lower	Upper	*P-Value*	AOR	Lower	Upper	*P-Value*
Marital status								
Single	Ref							
Married	0.366	0.210	0.639	<.001	0.282	0.022	3.668	.334
Divorced	0.628	0.234	1.686	.356	1.374	0.401	4.713	.613
Widowed	1.420	0.395	5.098	.591	3.921	0.790	19.467	.095
Occupation								
Housewife	Ref							
Farmer	0.411	0.200	0.844	.016	1.786	0.293	10.889	.529
Employed	0.000	0.000	-	.998	0.810	0.156	4.196	.801
Business	0.500	0.088	2.841	.434	0.000	0.000	-	.998
Marriage Duration								
1 to 5	Ref							
6 to 10	0.455	0.209	0.988	.046	0.488	0.220	1.084	.078
11 and above	1.071	0.548	2.094	.840	1.196	0.598	2.391	.613
Not applicable	2.065	1.097	3.887	.025	0.602	0.043	8.450	.706
Attitude								
Negative	Ref							
Positive	4.212	2.486	7.135	<.001	2.307	1.243	4.281	.008
Perceived Subjective Norm								
Low	Ref							
High	1.967	1.021	3.792	.043	2.074	0.897	4.797	.088
Perceived Behavior Control								
Negative	Ref							
Positive	9.306	4.338	19.963	<.001	6.015	0.017	2.569	<.001
Intention								
Low	Ref							
High	0.041	0.021	0.082	<.001	0.038	0.018	0.080	<.001

## DISCUSSION

The study findings show that 78% of women of childbearing age used one of the five identified modern family methods. This is comparable with the TDIHS data that showed that the uptake of modern family planning is increasing in Malinyi DC as well as the Morogoro region. ^2^ The most commonly used method is implants, which has been scaled to 54.1%. Condom use as an MFP method was relatively low among women of childbearing age, with only 2.8% of respondents choosing condoms over other MFP methods. Condoms are revealed in a low percentage because of the beliefs about the effect of condoms. It is believed the oil in the condom can cause cancer. The low prevalence can also be explained by the refusal by the partner, lack of reliability, and convenience of carrying.

In this study, the majority of women, 342 (81%), had a positive attitude, a high perceived subjective norm of 68.6% (n = 289), positively perceived behavioural control of 388 (92%), and a high intention to use modern family planning 361 (85.7%). These findings were consistent with the previous studies done in rural Uganda ^20^, North Kalimantan ^21^, Indonesia ^17^, South East Wales ^22^ and Ghana. ^23^ The main reason for their similarity could be better explained by the availability of family planning services and reproductive health education, particularly family planning, which has played a crucial role in raising the community awareness and uptake of family planning. In this study, healthcare facilities were the main primary source of information about MFP, 86.9% (n=284), which is also supported by the findings from the previous study. ^18^

The study revealed that more than 85% of women of reproductive age had high intentions to use modern family planning, but surprisingly, high intention to use modern family planning (AOR 0.038: 95% CI; 0.018 to 0.080) was less likely to be statistically significantly associated with increased uptake of modern family planning, which was also similarly reported in the study conducted in rural Uganda.^20^ These findings were not in agreement with the study conducted in Indonesia, North Kalimantan, and East Java, which reported a strong positive association between high intention and the use of modern family planning.^17,21,24^

The reason for this discrepancy could be due to a number of circumstances, including societal, political, and economic constraints that go beyond an individual’s control and impede women’s access to and use of contraceptives, even in the case that they have strong intentions to use them. Moreover, the intention of using MFP was directly found to be associated with attitude, perceived subjective norms, and perceived behavioural control. The similar finding was also reported in the previous studies conducted in North Kalimantan, Ghana, and East Java.^21,23,24^

This study revealed that a positive attitude, a high perceived subjective norm, and positively perceived behavioural control toward uptake of modern family planning have a direct influence on a high intention of using MFP. The binary regression model revealed a positive attitude and high perceived behavioural to have a positive direct effect on the uptake of MFP. However, the behavioural intention was found to have a negative influence. Therefore, the theory of planned behaviour is better at predicting the intention of behaviour.

This was a cross-sectional quantitative study using a population of women of reproductive age; the results should be interpreted with this limitation in mind. Future studies may consider including married men and using a mixed-methods design, including a qualitative component that may help explain the results.

## CONCLUSION

Positive attitude and high perceived behavioural control have a direct positive effect on the uptake of modern family planning. The family planning education-based programs could be offered to the community, particularly in rural areas, to maximise the community awareness and uptake of modern family planning. The theory of planned behaviour could predict intention to use but not the actual utilisation of modern family planning. A combination of theoretical models may be required to understand additional external elements that may influence the utilisation of modern family planning.
